# Accident vasculaire cérébral ischémique révélant une maladie coeliaque

**DOI:** 10.4314/pamj.v5i1.56196

**Published:** 2010-04-11

**Authors:** Belfkih Rachid, Souirti Zouhayr, Naima Chtaou, Ouafae Messouak, Faouzi Belahsen

**Affiliations:** 1 Service de Neurologie, CHU Hassan II, Fès, 30 000, Maroc

**Keywords:** maladie caeliaque, Accident vasculaire cérébral ischémique, maladie caeliaque, Maroc

## Abstract

La maladie cœliaque (MC) de l’adulte est une pathologie fréquente dont la présentation clinique est polymorphe. Les manifestations extradigestives sont multiples et rendent le diagnostic difficile lorsqu’elles sont isolées. Nous rapportons le cas d’un patient de 52 ans qui présentait un accident vasculaire cérébral ischémique (AVCI). Le bilan étiologique objectivait une hyperhomocystéinémie avec une carence en vitamine B12. La biopsie duodénale était en faveur d’une maladie coeliaque. Les anticorps antigliadines étaient positifs. Le patient fût mis sous régime sans gluten, antiaggrégant plaquettaire et hydroxocobalamine avec une évolution favorable.

## Background

La maladie coeliaque est une entéropathie inflammatoire chronique auto-immune provoquée par un antigène alimentaire la gliadine du gluten. Les aspects neuropathologiques sont hétérogènes, les mécanismes impliqués sont mal connus [1]. Des accidents vasculaires cérébraux ischémiques ont été très rarement décrits dans ce contexte. Nous en rapportons un cas.

## Patient et observation

Patient de 52 ans, sans antécédent pathologique notable, qui présenta le 20/04/09, de façon brutale un trouble de la parole et de la déglutition sans signes d’hypertension intracrânienne ni trouble de la vigilance dans un contexte d’apyrexie et de conservation de l’état général. L’examen général trouva un patient conscient, apyrétique à 37° C, une TA à 150/80 mmHg. L’examen neurologique objectiva un un syndrome pseudobulbaire fait de pleurers et rires spasmodiques, un trouble de la sensibilité profonde vibratoire et du sens de position du gros orteil des 2 membres inférieures, des réflexes ostéo-tendineux et cutanés conservés, sans autre déficit moteur ni trouble de la coordination ni trouble des fonctions supérieures. L’examen des autres appareils était sans particularité.

**Figure 1:**
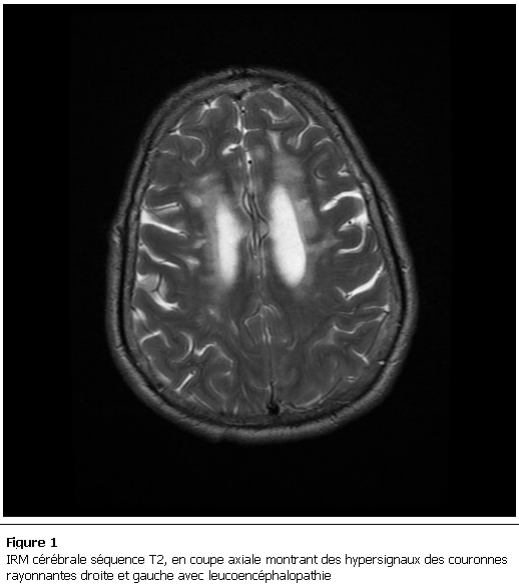
IRM cérébrale séquence T2, en coupe axiale montrant des hypersignaux des couronnes rayonnantes droite et gauche avec leucoencéphalopathie.

**Figure 2:**
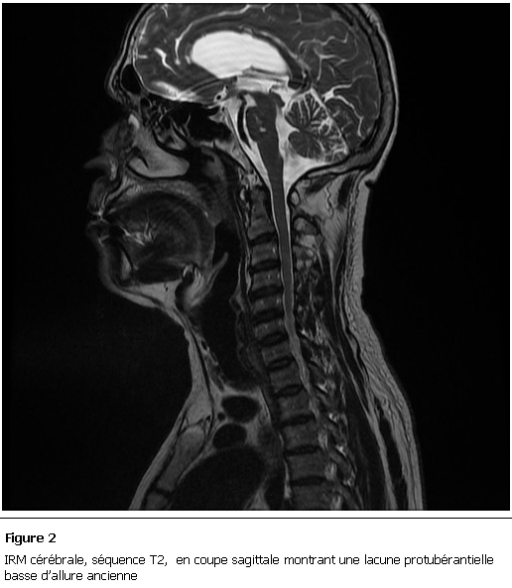
IRM cérébrale, séquence T2, en coupe sagittale montrant une lacune protubérantielle basse d’allure ancienne.

Le scanner cérébral montra des images lacunaires siégeant au niveau des 2 hémisphères cérébraux en sous cortical. L’imagerie par résonance magnétique cérébrale objectiva, à l’étage sus tentoriel, des anomalies de signal de la substance blanche périventriculaire diffuse en plages en hypersignal T2 et FLAIR et en séquence de diffusion ne prenant pas le produit de contraste signant la nature ischémique. A l’étage sous tentoriel, on retrouve des anomalies de signal au niveau bulbaire et de l’hémisphère cérébelleux gauche en hyper T2 et FLAIR non visible en diffusion et non modifiées après injection du gadolinium dont l’origine pourrait être ischémique ou inflammatoire (figure 1, 2). L’IRM médullaire était sans anomalies. L’étude du liquide céphalo-rachidien (LCR) après ponction lombaire était normale. L’electro cardiogramme (ECG), l’écho-doppler des troncs supra-aortiques, l’échographie doppler cardiaque transthoracique et le bilan lipidique étaient normaux. L’hémogramme était normal avec un taux d’hémoglobine à 14 g/dl, l’ionogramme sanguin, le bilan thyroïdien, le bilan hépatique étaient sans anomalie. Les sérologies des hépatites, de l’HIV et borréliose étaient négatives. Par ailleurs la vitamine B12 et l’acide folique étaient diminués avec des taux successifs de 124 pg/ml et 1,54ng/ml avec une hyperhomocystéinémie à 17 micro-mol/l. La fibroscopie oeso-gastro-duodénale avec biopsies fundique, antrale et jéjunale était en faveur de la maladie coeliaque. Les anticorps anti-facteur intrinsèque et anti-cellule pariétale étaient négatifs. Par contre les anti-gliadines et anti-endomysium étaient fortement positifs. La recherche des anticorps anti-nucléaires et anti-DNA natifs était négative.

Le patient fût mis sous régime sans gluten, hydroxocobalamine injectable et anti-aggrégant plaquettaire pour prévention secondaire de son AVC ischémique. L’évolution était favorable avec un recul de 6 mois.

## Discussion

La MC est une pathologie fréquente, dont la prévalence est estimée à 1% en Europe. La sex-ratio est de deux femmes pour un homme. La maladie est généralement découverte lors de la quatrième ou cinquième décennie [2].

La physiopathologie de la MC fait intervenir plusieurs éléments. Les deux antigènes principaux sont la gliadine, fraction protéique des céréales (seigle, blé, orge) et surtout la transglutaminase tissulaire (tTG) qui permet notamment la transformation des résidus glutamines de la gliadine en glutamates [3]. Celles-ci favorisent la liaison de la gliadine aux antigènes HLA présents dans la quasi-totalité des cas de MC, le HLA DQ2, dans plus de 90% des cas et DQ8 [4]. La présentation des antigènes dérivés de la gliadine par les cellules dendritiques déclenche une réponse immunitaire adaptative cellulaire et humorale entrainant la lyse des cellules épithéliales digestives responsables des manifestations cliniques de la maladie [5].

Les manifestations cliniques de la MC rendent compte de plusieurs phénomènes: atteinte digestive, atteintes extradigestives, complications ou pathologies associées à la MC. En effet, la MC est associée à de nombreuses endocrinopathies (diabète de type I, thyroïdite), à des manifestations cutanées (dermatite herpétiforme), à des syndromes malformatifs, notamment la trisomie 21, au déficit en IgA et à diverses manifestations neurologiques [2]. La confirmation diagnostique est basée sur les sérologies et la biopsie duodénale

Les atteintes neurologiques seraient présentes dans 10% des cas. La survenue d’AVCI au cours de la MC est rare.

L’hyperhomocystéinémie, secondaire à une carence en vitamine B12 et en acide folique, est un facteur de risque reconnu dans la survenue d’accidents ischémiques cérébraux. Plusieurs études contrôlées ont démontré un risque d’AVC ischémique multiplié par deux lorsqu’il existe une hyperhomocystéinémie pathologique [5]. Récemment, l’hypothèse d’une angiopathie cérébrale a été évoquée dans la genèse de certaines manifestations neurologiques de la MC. En effet, la tTG est présente dans le tissu cérébral où elle joue un rôle important dans le maintien de l’intégrité endothéliale et dans le métabolisme des Cellules neuronales. Les anticorps IgA anti-tTG dirigés contre la TG endothéliale pourraient être responsables d’une angiopathie cérébrale auto-immune à l’origine de l’AVC ischémique [6,7].

Devant l’âge jeune de notre patient, l’absence de facteur de risque vasculaire, l’absence de cause cardiaque, la négativité du reste du bilan inflammatoire et immunologique plaident en faveur de l’existence d’un lien de causalité entre l’accident vasculaire cérébral ischémique (AVCI) et la maladie coeliaque (MC). Concernant l’implication des antiphospholipides : une étude cas témoin récente n’a pas montré de différence significative dans la prévalence des anticorps anticardiolipines et anti-ß2GPI, hormis pour les anticorps anticardiolipines d’isotype IgA, dont la fréquence semble plus élevée au cours de la MC [8]. Cependant, aucun de ces patients ne présentait de manifestations thrombotiques.

## Conclusion

La maladie coeliaque est une affection à symptomatologie polymorphe dont le diagnostic doit êrtre évoqué devant de nombreuses manifestations cliniques extradigestives parmi lesquelles l’accident vasculaire cérébral ischémique de l’adulte jeune d’où l’intérêt de rechercher une MC devant les cas d’AVC ischémiques inexpliqués de l’adulte jeune.

## Conflits d’intérêts

Les auteurs ne déclarent aucun conflits d’intérêts.
